# The Essential Functions and Detection of Bisecting GlcNAc in Cell Biology

**DOI:** 10.3389/fchem.2020.00511

**Published:** 2020-07-03

**Authors:** Qiushi Chen, Zengqi Tan, Feng Guan, Yan Ren

**Affiliations:** ^1^Clinical Laboratory of BGI Health, BGI-Shenzhen, Shenzhen, China; ^2^Joint International Research Laboratory of Glycobiology and Medical Chemistry, College of Life Sciences, Northwest University, Xi'an, China; ^3^Institute of Interdisciplinary Integrative Medicine Research, Shanghai University of Traditional Chinese Medicine, Shanghai, China

**Keywords:** bisecting GlcNAc, mass spectrometry, glycosylation, N-glycan, GlcNAc-T III

## Abstract

The N-glycans of mammalian glycoproteins vary greatly in structure, and the biological importance of these variations is mostly unknown. It is widely acknowledged that the bisecting N-acetylglucosamine (GlcNAc) structure, a β1,4-linked GlcNAc attached to the core β-mannose residue, represents a special type of N-glycosylated modification, and it has been reported to be involved in various biological processes, such as cell adhesion, fertilization and fetal development, neuritogenesis, and tumor development. In particular, the occurrence of N-glycans with a bisecting GlcNAc modification on proteins has been proven, with many implications for immune biology. Due to the essential functions of bisecting GlcNAc structures, analytical approaches to this modification are highly required. The traditional approach that has been used for bisecting GlcNAc determinations is based on the lectin recognition of *Phaseolus vulgaris* erythroagglutinin (PHA-E); however, poor binding specificity hinders the application of this method. With the development of mass spectrometry (MS) with high resolution and improved sensitivity and accuracy, MS-based glycomic analysis has provided precise characterization and quantification for glycosylation modification. In this review, we first provide an overview of the bisecting GlcNAc structure and its biological importance in neurological systems, immune tolerance, immunoglobulin G (IgG), and tumor metastasis and development and then summarize approaches to its determination by MS for performing precise functional studies. This review is valuable for those readers who are interested in the importance of bisecting GlcNAc in cell biology.

## Introduction

The monosaccharide-amino acid linkage of N-acetylglucosamine (GlcNAc) β1- asparagine (Asn) was originally discovered in biochemical analyses of abundant glycoproteins present in serum, e.g., immunoglobulins (Imperiali and Hendrickson, 1995; Cobb, 2020). Since then, glycans that covalently attached to proteins at Asn residues by an N-glycosidic bond have been termed N-glycans. This attachment usually occurs in a conserved sequence Asn-X-Ser/Thr, in which X can be any amino acid except proline (Pro) (Varki, 2009; Taylor and Drickamer, 2011; Chung et al., 2017).

**Figure 1:**
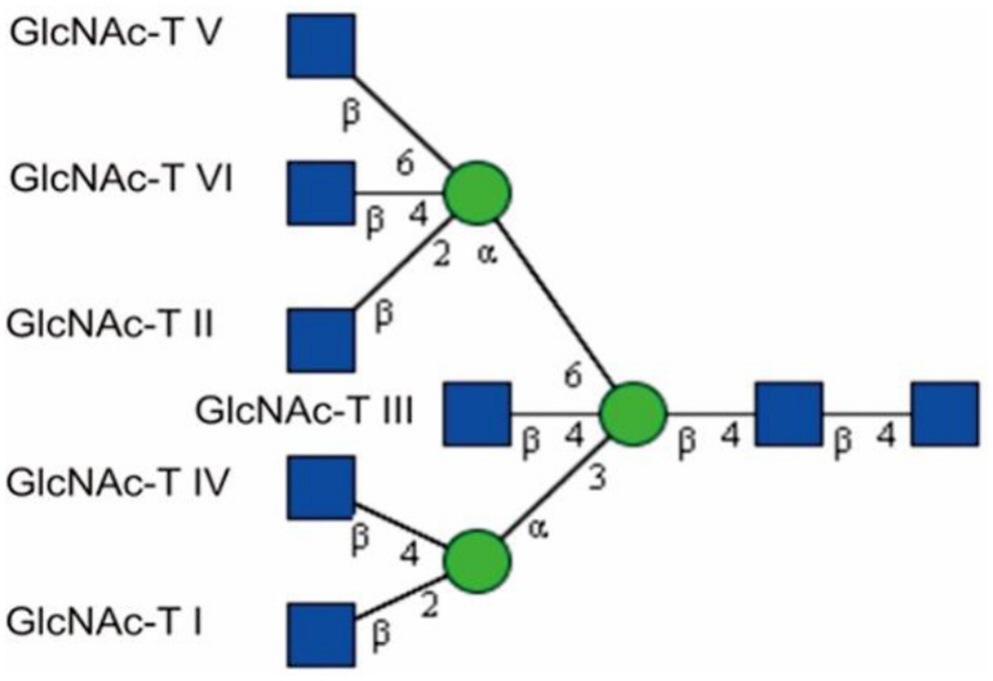
GlcNAc-Man branches catalyzed by GlcNAc-Ts, adapted from Chen (2015) with permission from Qiushi Chen. The first antenna is initiated via the enzyme GlcNAc-T I. GlcNAc-T II creates a biantennary glycan, and GlcNAc-T III yields a bisecting GlcNAc. More branches can be produced via the action of GlcNAc-T IV, V, and VI. 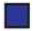 GlcNAc, 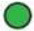 Man.

A distinctive structural feature of N-glycans is the presence of several GlcNAc antennae (branches) that are sequentially synthesized by a series of Golgi-resident glycosyltransferases, N-acetylglucosaminyltransferases (GlcNAc-Ts) (Figure 1) (Schachter, 1991; Kizuka and Taniguchi, 2018). N-glycans can be divided into three categories: high-mannose, hybrid, and complex. Hybrid and complex N-glycans may carry a bisecting GlcNAc group, which forms a new subtype of glycan termed bisecting GlcNAc (Harpaz and Schachter, 1980; Varki, 2009; Nakano et al., 2019). The discovery of this structure lagged behind the detection of other glycan structures due to the limitations of the detection approaches and the peculiarity of its structure. This type of glycan was reported in the 1970s and was detected by a combination of sequential exoglycosidase digestion, methylation derivatization, acetolysis, and Smith degradation from ovalbumin (Yamashita et al., 1978; Nagae et al., 2020). GlcNAc transferred to the 4-position of the β-linked core mannose (Man) residue in complex or hybrid N-glycans by the β1,4-mannosyl-glycoprotein 4-β-N-acetylglucosaminyltransferase (GlcNAc-T III) is considered as a bisecting structure that is usually not considered as an antenna because it cannot be further extended by the proper enzymes (Narasimhan, 1982; Schachter, 1991; Varki, 2009; Miwa et al., 2012; Chen et al., 2016). GlcNAc-T III is encoded by the gene *mgat3*, which was initially discovered from hen oviducts in 1982 (Narasimhan, 1982; Miwa et al., 2012). It has been reported that its distribution in human tissues is mainly in the brain, liver, placenta, bone marrow, and kidney (Nishikawa et al., 1992; Yoshimura et al., 1995b; Taniguchi et al., 1999; Takamatsu et al., 2004; Schedin-Weiss et al., 2019). So far, there are no reports on any tissue specificity that is related to the functions of this subtype of glycan. The addition of this GlcNAc requires the prior action of GlcNAc-T I (Schachter, 1991; Nakano et al., 2019). The existence of a bisecting GlcNAc prevents α-mannosidase II from trimming and has been proved to inhibit the activities of GlcNAc-T II, GlcNAc-T IV, and GlcNAc-T V *in vitro* as well (Schachter, 1991, 2014; Varki, 2009; Nakano et al., 2019). The addition of bisecting GlcNAc confers unique lectin recognition properties to this new subtype of glycan (Miwa et al., 2012; Nagae et al., 2013; Link-Lenczowski et al., 2018). B16 mouse melanoma transfected by *mgat3* that encodes GlcNAc-T III shows weaker binding to phytohemagglutinin-L (PHA-L) but stronger binding to *Phaseolus vulgaris* erythroagglutinin (PHA-E). The lectins of PHA-L and PHA-E show specific recognition to multiple antennary glycans and bisecting GlcNAc structures, respectively (Yoshimura et al., 1995c; Varki, 2009; Liu et al., 2016; Wu et al., 2019). This suggests that increased expression of GlcNAc-T III may result in a decrease in multiple branched N-glycan structures. The balance among different types of glycans may play an important role in controlling cell functions.

The N-glycans of mammalian glycoproteins vary greatly in structure, but the biological importance of these variations is mostly unknown (Bhattacharyya et al., 2002; Reily et al., 2019). It is widely acknowledged that bisecting GlcNAc represents a special type of N-glycosylated modification that is involved in various biological processes, such as cell adhesion, fertilization and fetal development, neuritogenesis, and tumor metastasis and development (Bhattacharyya et al., 2002; Kariya et al., 2008; Akasaka-Manya et al., 2010; Gu et al., 2012; Allam et al., 2015; Zhang et al., 2015; Kizuka and Taniguchi, 2018). The clearly altered levels of bisecting GlcNAc on integrin β1 have been reported to be responsible for early spontaneous miscarriages in humans (Zhang et al., 2015). Tan et al. found that bisecting GlcNAc is able to inhibit hypoxia-induced epithelial-mesenchymal transition in breast cancer cells (Li et al., 2016; Tan et al., 2018). The presence of glycoproteins bearing complex N-glycans with bisecting GlcNAc, fucose (Fuc) and N,N-diacetyllactosamine (LacdiNAc) structures was detected in extracellular vehicles (EVs) from ovarian carcinoma cells; however, the prevention of N-glycosylation processing from high mannose to complex glycans by kifunensine resulted in alterations in the components of EVs and triggered a decrease in several glycoproteins (Gomes et al., 2015). It has also been reported that the occurrence of a bisecting GlcNAc on glycoproteins has many implications in immune biology (el Ouagari et al., 1995; Yoshimura et al., 1996; Takegawa et al., 2005; Pang et al., 2007; Clark, 2014; Chen et al., 2016; Shade et al., 2019). For instance, K562 cells are easily killed by natural killer (NK) cells; however, after being transfected with *mgat3*, K562 cells acquired NK-cell resistance (Yoshimura et al., 1996; Patankar et al., 1997; Miwa et al., 2012). Therefore, it is very important to take a step forward and review this type of N-glycan. Although the method that is usually used in many studies for bisecting GlcNAc determination is lectin recognition by PHA-E, there are drawbacks to this method. The first disadvantage is low specificity and sensitivity (Dang et al., 2019). This is quite common in most of the lectin-glycan recognition methods. For instance, *Sambucus nigra* (elderberry) agglutinin (SNA) IV prefers to bind with α2,6-linked sialic acid but also has some binding to α2,3-linked sialic acid (Chen, 2015; Shang et al., 2015; Lis-Kuberka et al., 2019). The second drawback is that lectin recognition could not tell the relative amount of the bisecting GlcNAc structure. Last but not least, this method is not able to reveal the glycosylation site or the glycan structure. Instead, approaches based on mass spectrometry (MS) have been revealed in recent studies to be a suitable tool for expeditiously and precisely investigating this type of glycan.

MS is a technique that measures the mass-to-charge ratios of ions and has been used for small-molecule analysis since World War I (Calvete, 2014). It has a history of playing an important role in glycan or glycan-related studies since the 1960s. In 1968, electron ionization was used in the structural elucidation of di- and tri-saccharides (Kochetkov et al., 1968; Chen, 2015). At that time, it was difficult to detect more complex oligosaccharides since only the molecules possessing higher volatility were analyzable; however, more oligosaccharides in the complex glycans led to decreased volatility. Additionally, the mass range of MS detection restricted study of the complex oligosaccharides with higher molecular weights. In the late 1970s, MS was used for the first time in the study of blood glycoproteins from Antarctic fish, in which a proline-containing glycopeptide that had a disaccharide was sequenced; the structure of this disaccharide was identified as galactosyl-N-acetylgalactosamine (Morris et al., 1978; Dell and Morris, 2001; Bielik and Zaia, 2010). Several years later, Dell and Morris performed the first structural analysis of glycans using a fast-atom-bombardment mass spectrometer (FAB MS) (Dell et al., 1983; Dell and Morris, 2001). With its rapid development, MS has now significantly improved in its analytical scope, speed, and depth. For glycopeptide or glycan structure analysis, the Orbitrap mass spectrometers with higher resolution and accuracy are a good choice under MSn (n > 2) mode coupled with or without liquid chromatography (LC). Matrix-assisted laser desorption ionization time-of-flight (MALDI-TOF/TOF) MS with higher collision energies is also a valuable choice for MS1/MS2 analysis of glycans (Chen, 2015; Chen et al., 2016). Electrospray ionization (ESI)-TOF MS has also been reported to be efficient for glycosylation analysis of intact IgG molecules (Wei et al., 2019).

## The Functions of Bisecting GlcNAc Modification

### In Neurological Systems

Akasaka-Manya et al. discovered that the mRNA levels of *mgat3* were elevated in the temporal cortex of the brain in patients with Alzheimer's disease (AD) (Akasaka-Manya et al., 2010), which accelerated studies on the tissue distribution of GlcNAc-T III expression, with the conclusion that it was most highly expressed in the nervous system (Shimizu et al., 1993; Kizuka et al., 2016b; Kizuka and Taniguchi, 2018).

**Figure 2:**
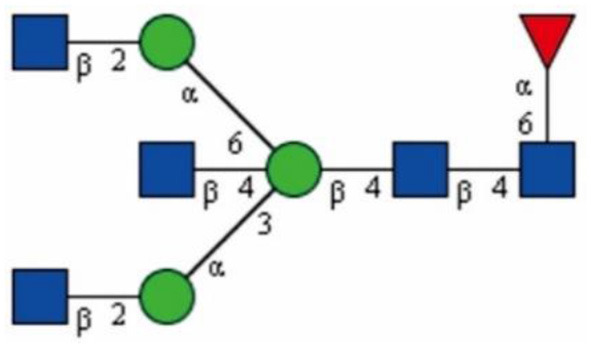
The bisecting GlcNAc structure proposed by Shimizu et al., drawn based on the information obtained from Shimizu et al. (1993). The proposed linkage between each monosaccharide is labeled. 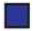 GlcNAc, 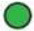 Man. 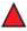 Fuc.

In 1993, Shimizu et al. reported that the main glycan structures detected in the mouse cerebrum, cerebellum, and brain stem are bisected, as is proposed in Figure 2 (Shimizu et al., 1993; Nagae et al., 2016). Later, Shigeta et al. found that GlcNAc-T III promoted β1 integrin-mediated neuritogenesis triggered by serum deprivation in Neuro2a cells and that the neuritogenesis induced by GlcNAc-T III was functionally blocked by anti-β1 integrin monoclonal antibody (DF5) (Shigeta et al., 2006). In addition, β1 integrin is found to be regulated as a target protein by GlcNAc-T III, and this could be supported by a study showing that the amount of β1 integrin in erythroagglutinating-phytohemagglutinin (E4-PHA)-associated complexes significantly increased in GlcNAc-T III transfectants compared with that in mock transfectants (Shigeta et al., 2006).

AD is a progressive, neurodegenerative disease in which there are deficits in memory and cognitive functions; more importantly, it is a global health problem (Abbott, 2011; Scheltens et al., 2016; Kizuka and Taniguchi, 2018; Schedin-Weiss et al., 2019). However, current treatments are still only symptomatic (Winblad et al., 2016; Schedin-Weiss et al., 2019). It is necessary to understand that many solid studies support connections between AD and aberrant protein glycosylation, considering the fact that glycoproteins including tau, Aβ-precursor protein (APP), and β-site APP-cleaving enzyme-1 (BACE-1) are involved in AD pathogenesis and have been found to show altered glycosylation patterns (Halim et al., 2011; Schedin-Weiss et al., 2014, 2019; Kizuka et al., 2015). More importantly, APP and BACE-1 contain glycosylation modifications with bisecting GlcNAc structures (Akasaka-Manya et al., 2008, 2010; Schedin-Weiss et al., 2014; Kizuka et al., 2015, 2016a). Bisecting GlcNAc modifications have shown the capacity to stabilize BACE1 protein under conditions of oxidative stress (Kizuka et al., 2016a), and the increased contents of bisecting GlcNAc in AD brains might function as an adaptive response, which protects the brains from the damage caused by additional beta-amyloid yields (Akasaka-Manya et al., 2010). One study showed that the lack of bisecting GlcNAc to BACE1 directed the transport of this protein to the lysosome and accelerated its degradation, which resulted in the less accumulation of β amyloid in AD (Kizuka et al., 2015; Kizuka and Taniguchi, 2018). These findings have highlighted the importance of bisecting GlcNAc modification in the nervous system. However, the underlying mechanism is still not clear.

AD is a chronic disease and begins to develop decades before the first symptoms appear, which suggests the importance of investigating early changes (e.g., glycan alteration) for improving early diagnosis. We have recently published our findings on glycosylation changes in AD research. An increase in bisecting N-GlcNAc modifications was observed in cerebrospinal fluid (CSF) from AD patients. The further investigation of CSF from 242 patients with subjective cognitive impairment (SCI), mild cognitive impairment (MCI), or AD revealed more glycoproteins binding to PHA-E in MCI and AD than in SCI (Schedin-Weiss et al., 2019). Therefore, these findings could be essential for developing early AD diagnosis biomarkers and understanding the early stages of AD development, which might be additionally beneficial for designing novel AD treatment strategies. The challenges in the future will be to perform comprehensive and detailed glycoproteomic and glycomic analysis of those glycoproteins with bisecting GlcNAc modification.

### In Immune Tolerance

In 1996, Clark et al. proposed the human fetoembryonic defense system hypothesis (hu-FEDS) (Clark et al., 1996; Pang et al., 2016). The basic concept of this hypothesis is that glycoproteins expressed in the reproductive system and gametes can either inhibit immune responses or prevent rejection. Indeed, glycoproteins in human seminal plasma and the pregnant uterus have been shown to suppress immune responses *in vitro*, specifically for those glycoproteins containing bisecting GlcNAc structures (Bolton et al., 1987; Kelly and Critchley, 1997; Clark, 2014; Szczykutowicz et al., 2019).

Bisecting GlcNAc structures have been reported to possess immune suppression functions. For instance, K562 cells are easily killed by natural killer (NK) cells; however, after being transfected with the gene that encodes GlcNAc-T III, K562 cells possessing more bisecting GlcNAc attain NK cell resistance (Yoshimura et al., 1996; Patankar et al., 1997). Natural killer (NK) cells are the major type of immune cells found in the human uterus, which indicates that they potentially target sperm (King et al., 1991; Clark, 2014). Human sperm were found to express bisecting GlcNAc structures, which explains why sperm are not killed by the maternal immune system when entering the female as a foreign substrate and thus support hu-FEDS (Pang et al., 2007; Clark, 2014; Szczykutowicz et al., 2019). Additionally, abundant bisecting GlcNAc glycans were detected in human syncytiotrophoblasts (STB) and cytotrophoblasts (CTB) (Chen et al., 2016). It is most likely that the maternal immune system was suppressed due to the presence of bisecting GlcNAc glycans and that the fetus benefited from this suppression; the mother could nourish a fetus (similar to a foreign organ as the father contributes to its half genome) within her body for several months without rejection. The possible mechanism underlying this suppression could be that the glycoconjugates interacted with lectins that linked to particular signal transduction pathways modulating immune cell functions. For instance, α-2,3-linked sialic acid on soluble CD52, a glycoprotein of 12 amino acids anchored to glycosylphosphatidylinositol, could mediate T-cell suppression by binding to siglec-10 (Clark, 2014; Shathili et al., 2019). It is possible that bisecting GlcNAc can function in a similar way to suppress NK cells.

### On Immunoglobulin G (IgG)

IgG is an important molecule in the immune system. IgG regulates its immune functions through complement and cellular IgG-Fc gamma receptors (FcγR) (Dekkers et al., 2016). It contains a highly conserved N-linked glycan at position Asn297 in the Fc region (Arnold et al., 2007; Dekkers et al., 2016; Kiyoshi et al., 2017). This glycan is composed of variable levels of fucose, galactose, and sialic acid and bisecting GlcNAc (Le et al., 2016; Gudelj et al., 2018; Lu and Holland, 2019; Shade et al., 2019). It is widely acknowledged that the Fc-glycan has an influence on the biological activities of IgG. For example, a lack of fucose in the Fc glycan significantly improves binding to the human FcγR III, and this result is applied to improve the efficacy of therapeutic monoclonal antibodies. Attachment of bisecting GlcNAc to the Fc glycan has been described to induce antibody-dependent cell-mediated cytotoxicity (ADCC) (Shields et al., 2002; Hodoniczky et al., 2005; Dekkers et al., 2016).

Studies focused on characterizing the IgG- and IgA-linked glycans have shown that glycans are differentially expressed in the setting of autoimmunity. For instance, patients <50 years old with Lambert-Eaton myasthenic syndrome (an autoimmune disease in which the immune system attacks the body's own tissues) show increased levels of bisecting GlcNAc on IgG1 and IgG2 (Selman et al., 2011; Maverakis et al., 2015). This suggests that particular glycan types may be potential biomarkers for certain diseases.

### In Tumor Metastasis and Development

It is essential to understand the factors that affect tumor progression so as to determine how to control tumor growth and metastasis. It has been reported that more multiple branched N-glycan modifications occur in tumor cells due to the higher activity of GlcNAc-T V, which promotes tumor cell metastasis (Dennis et al., 1987; Gu et al., 2009; Kizuka and Taniguchi, 2016). A possible explanation for this is that β1,6-GlcNAc-branched N-glycans can be preferentially processed by β1,4 Gal-T, and β1,3 GlcNAc-T to form poly-N-acetyllacotosamine (poly-LacNAc) for elongation of N-glycans, which could be further modified into the motifs involved in cancer metastasis, such as sialyl Lewis X (Yamadera et al., 2018). As mentioned above, the increased expression of GlcNAc-T III prevented the formation of multiple branch glycans, as GlcNAc-T V could not extend the glycans beyond the bisecting GlcNAc structure and thus inhibited tumor cell metastasis (Dennis et al., 1987; Gu et al., 2009; Taniguchi and Korekane, 2011). Two years ago, it was reported that bisecting GlcNAc structures could inhibit hypoxia-induced epithelial-mesenchymal transition in breast cancer (Tan et al., 2018). However, the mechanism underlying is still not clear. It has been speculated that the addition of bisecting GlcNAc to the key glycoproteins of signaling transduction, e.g., growth factors, integrins, and cytokine receptors, has its special signaling strength under hypoxia. Actually, observations of non-solid tumors contradict this explanation. GlcNAc-T III was more activated in patients with chronic myelogeneous leukemia in blast crisis (CML-BC) and in patients with multiple myeloma (MM) (Yoshimura et al., 1995a).

Alterations in glycosylation are usually considered as a hallmark of cancer, and the protein with the most extensive studies of its glycosylation is E-cadherin (de-Freitas-Junior et al., 2013). In 2019, researchers found that E-cadherin was required for metastasis in multiple breast cancer models (Padmanaban et al., 2019) and that it contained bisecting GlcNAc modifications (Kitada et al., 2001; de-Freitas-Junior et al., 2013). The addition of bisecting GlcNAc to E-cadherin was found to negatively regulate the tyrosine phosphorylation of β-catenin (Kitada et al., 2001; Takahashi et al., 2009), and GlcNAc T-III knockdown cells displayed a membrane delocalization of E-cadherin, resulting in its cytoplasmic accumulation (Pinho et al., 2009). As a result, the deactivated β-catenin failed to enhance cell growth or oncogenesis as it formed a tight complex with E-cadherin and could not be translocated into the nuclei (Gu et al., 2009). These results suggest that bisecting GlcNAc plays important roles in tumor metastasis and development. Therefore, it is reasonable that certain aberrant glycosylation (e.g., bisecting) patterns could be used as biomarkers for the progression of particular diseases, including cancer metastasis and development (Dennis et al., 1999; Tan et al., 2018).

### Others

It has been reported there are multiple functions of bisecting GlcNAc in other cell biology processes. The bisecting GlcNAc structure in N-glycans of adenylyl cyclase III was proved to be an enhancer of enzyme activity (Li et al., 2007). The bisecting GlcNAc structure has been found to inhibit stroma-dependent hemopoiesis in transgenic mice expressing GlcNAc-T III (Yoshimura et al., 1998).

## The Detection of Bisecting GlcNAc Structures

The approaches reviewed here have been released and proved as efficient tools for bisecting GlcNAc modification studies. These bisecting GlcNAc determination approaches are reviewed based on two detection targets, namely, glycan and glycopeptide levels. Before the samples are subjected to glycan or glycopeptide analysis, cell or tissue samples need to be processed as previously described (North et al., 2010; Chen, 2015; Chen et al., 2016); the procedure for sample preparation will not be addressed here.

### Approaches for Detecting Glycan Levels

#### β1,4-Galactosyltransferase Reaction

β1,4-galactosyltransferase is an enzyme that transfers a galactose (Gal) from UDP-Gal to GlcNAc and forms the disaccharide unit of Galβ1,4GlcNAc in the antenna of complex and hybrid glycans (Schwientek et al., 1996; Chen et al., 2016). However, if a GlcNAc is at the bisected position, it will not be processed by this enzyme, and there are no changes for glycans containing a bisecting GlcNAc (Pang et al., 2007; Qasba et al., 2008;Chen, 2015).

**Figure 3:**
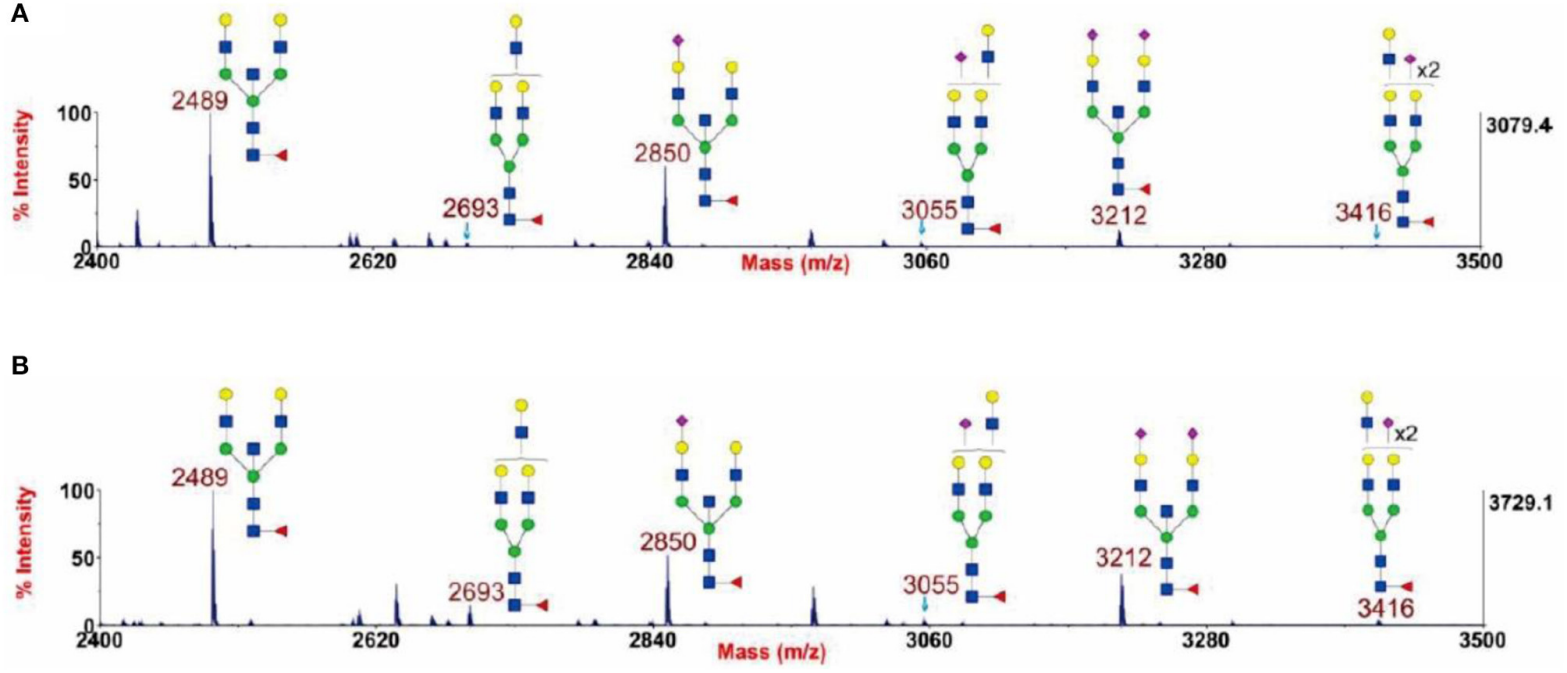
Annotated MALDI-TOF MS spectra of permethylated N-glycans **(A)** and permethylated β1,4-galactosyltransferase treated N-glycans **(B)** from human cytotrophoblasts (CTB), adapted from Chen et al. (2016) with permission from Qiushi Chen. 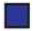 GlcNAc, 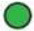 Man, 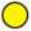 Gal, 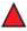 Fuc, 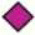 NeuAc.

In our previous study, we adopted this method to prove the presence of a bisecting GlcNAc structure in glycans through the β1,4-galactosyltransferase reaction, as is shown in Figure 3 (Chen et al., 2016). Using this strategy, the glycan sample was treated by the enzyme at 37°C for 24 h to ensure a complete reaction (Chen, 2015). The glycans at m/z 2,489, 2,850, and 3,212 were chosen as the targets of observation because these glycans contain a potential substrate (an unmodified GlcNAc) for β1,4-galactosyltransferase. If the structures included a reactable GlcNAc group, a Gal residue would be incorporated into the structure, resulting in the glycans at m/z 2,489, 2,850, and 3,212 undergoing a shift in m/z to 2,693, 3,055, and 3,416, respectively. Figure 3 displays two typical spectra scanned by MALDI-TOF MS with or without β1,4-galactosyltransferase treatment. This figure clearly shows that after the enzyme treatment, there are no obvious changes in the three glycan comparison groups at m/z 2,489 and 2,693, 2,850 and 3,055, and 3,212 and 3,416. This result demonstrates that the GlcNAc in these three glycan structures cannot be extended by β1,4-galactosyltransferase and the bisecting GlcNAc present in these glycans.

The processing performed using this approach is quite simple, and the interpretation of the results is so direct that there is no need for any software for further data analysis. The signal alteration from glycans is basic and essential for this method. However, some exceptions have been observed in the application of this approach *in vitro*. A research article reported a successful galactosylation occurring beyond the bisecting GlcNAc in the structure of GlcNAcMan3GlcNAc2 *in vitro* (Zou et al., 2011). In addition, we found that a chemoenzymatically synthesized glycan structure (Galb1-4GlcNAcb1-2 Mana1-6(Galb1-4GlcNAcb1-4)(Galb1-4GlcNAcb1-2Mana1-3)Manb1-4GlcNAcb1-4(Fuca1-6)GlcNAc) containing galactosylated bisecting GlcNAc was clearly labeled for Functional Glycomics (CFG) array (CFG, 2012).

Therefore, it would be better to combine the approach of galactosyltransferase reaction with other methods listed in this review to confirm the presence of a bisecting GlcNAc structure in glycans. In 2016, we adopted this method together with gas chromatography-mass spectrometry (GC-MS) to study the bisecting GlcNAc modification (Chen et al., 2016), which will be described in the following section.

#### Gas Chromatography-Mass Spectrometry (GC-MS)

GC-MS methods adopted to detect bisecting GlcNAc have been described previously (Ciucanu, 2006; North et al., 2010). Considering the volatile analytes necessary for GC-MS detection, the glycan samples must be derivatized into partially methylated alditol acetates (PMAA) before being subjected to MS, as has been published in some reports (North et al., 2010; Chen, 2015): the permethylated glycan sample was treated with a NaBD_4_ solution and was then dried under nitrogen assistance, followed by acetylation treatment with acetic anhydride. As shown in Figure 1, the bisecting GlcNAc is directly attached to the C4 position of the β-linked Man, which possesses a characteristic component of 3,4,6-linked Man, which is a unique signal for the identification of bisecting GlcNAc by GC-MS.

**Figure 4:**
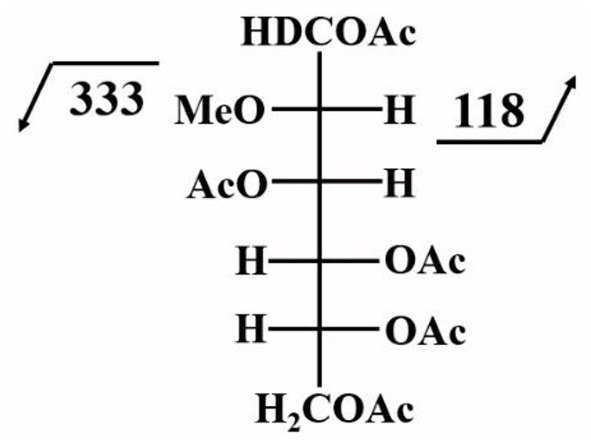
Characteristic fragment ions of the PMAA derivative of a 3,4,6-linked-D-mannopyranosyl residue; this figure is modified from CCRC (2020) with permission from CCRC.

Figure 4 shows the structural molecule of the PMAA derivative of a 3,4,6-linked-D-mannopyranosyl residue. The fragmentation of this molecule can yield two characteristic ions with m/z of 118 and 333.

We have adopted this permethylation method and combined GC-MS detection to prove the presence of bisecting GlcNAc in human CTB and STB (Chen et al., 2016). As shown in Table 1, the characteristic fragment ions of m/z 118 and 333 were simultaneously detected for the group of 3,4,6-linked Man, which supported the existence of bisecting GlcNAc.

**Table 1:** Summary of the GC-MS linkage analysis of partially methylated alditol acetates derived from N-glycans of cytotrophoblasts (CTB) and syncytiotrophoblasts (STB), adapted from Chen et al. (2016) with permission from Qiushi Chen.

**Elution time, min (CTB)**	**Elution time, min (STB)**	**Characteristic fragment ions**	**Assignments**	**Relative abundance (CTB)**	**Relative abundance (STB)**
16.95	16.90	102, 115, 118, 131, 162, 175	Terminal Fuc	0.16	0.14
18.45	18.40	102, 118, 129, 145, 161, 205	Terminal Man	0.68	0.62
18.71	18.67	102, 118, 129, 145, 161, 205	Terminal Gal	0.15	0.17
19.62	19.56	129, 130, 161, 190, 234	2-linked Man	1	1
19.90	19.85	118, 129, 161, 203, 234	3-linked Gal	0.07	0.10
21.18	21.14	87, 88, 129, 130, 189, 190	2,6-linked Man	0.05	0.05
21.34	21.30	118, 129, 189, 202, 234	3,6-linked Man	0.33	0.34
21.80	21.76	118, 139, 259, 333	3,4,6-linked Man	0.08	0.07
22.27	22.23	117, 129, 145, 205, 247	Terminal GlcNAc	0.04	0.04
23.15	23.12	117, 159, 233	4-linked GlcNAc	0.22	0.39
24.00	23.96	117, 159, 346	3,4-linked GlcNAc	0.03	0.03
24.46	24.42	117, 159, 261	4,6-linked GlcNAc	0.04	0.08

*The elution time is indicated in minutes, and the relative abundance of 2-linked mannose (major component) is normalized to 1*.

In this method, GC was used for the separation of analytes and it thus has higher resolution for complex small molecules; however, the glycan samples must be derivatized into PMAA for GC-MS analysis, and the reaction efficiency affects the quantification of the bisecting GlcNAc structures.

#### Multi-Stage Mass Spectrometry (MSn)

In principle, this method is quite similar to GC-MS detection because the detection of bisected glycan structures can be accomplished by identifying the presence of the 3,4,6-linked Man (Allam et al., 2015). In this method, the Obitrap MS was used for multiple fragmentation (Allam et al., 2015).

**Figure 5:**
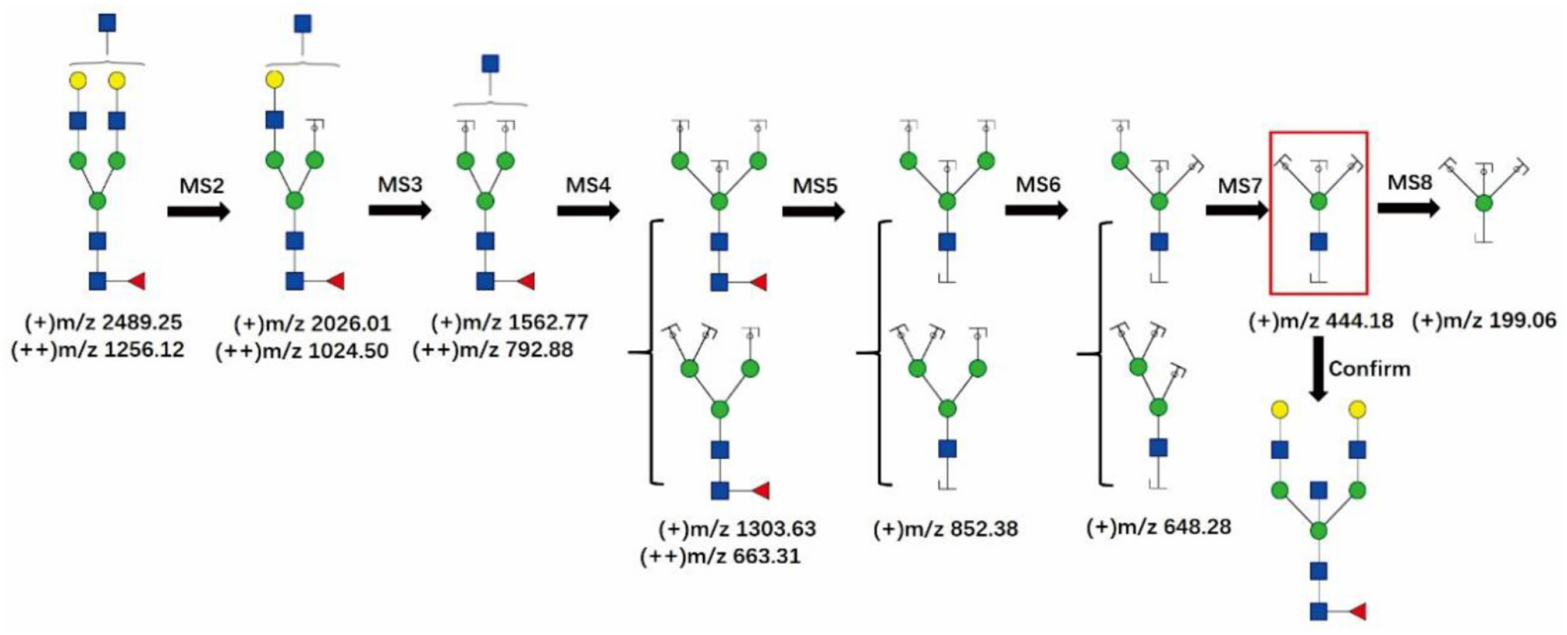
The MS8 approach for confirming the presence of bisecting GlcNAc structures. The fragment ion at m/z 444.18 in the red frame is the characteristic ion of the bisecting GlcNAc glycans. 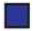 GlcNAc, 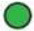 Man, 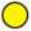 Gal, 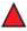 Fuc.

Figure 5 shows the logical order of the MS8 approach that was used for detecting bisecting GlcNAc in the glycan at m/z 2489.25, which is a bitennary, core-fucosylated glycan. Theoretically, the MS7 spectrum of the glycan at m/z 2489.25 should display the characteristic ion of the bisected glycan at m/z 444.18, which would support the presence of 3,4,6-linked Man. Additionally, MS8 analysis would be further carried out to show that the ion at m/z 444.18 is truly a glycan fragment ion indeed and is not noise or a contaminant.

This method is able to target the bisecting GlcNAc structure of interest. More importantly, it does not require additional sample processing. However, it is highly dependent on the MS analyzer, as well as on operator techniques. Usually, only glycans with higher abundances can provide good signals with multiple fragmentation under MSn mode.

### Approach for Detecting Glycopeptide Levels

Due to the rapid development of MS techniques, it is possible to perform analysis of glycopeptides composed of the peptides together with their glycans. In addition to detecting bisecting GlcNAc, MS can also confirm the glycosylation sites as well as the glycan components. The method introduced here for bisecting GlcNAc detection references the paper published in Analytical Chemistry in 2019 (Dang et al., 2019), which was designed to detect bisecting GlcNAc in glycopeptides by their characteristic ion(s) in fragmented MS/MS spectra under low-energy collisions. The characteristic ion(s) are either [Pep+HexNAc_3_Hex] or [Pep+FucHexNAc_3_Hex] or both. In this paper, 25 glycoproteins (possessing bisecting GlcNAc) were identified from rat kidney tissue, four of which (Q01129 decorin, P17046 lysosome-associated membrane glycoprotein 2, P07861 neprilysin, and B5DFC9 nidogen-2) were found to be protein analogs of those identified in our human amnion samples. More importantly, one of these glycoproteins, neprilysin, has the same bisecting GlcNAc location (site N285) as the human neprilysin (P08473) (data not shown).

This method can simultaneously obtain precise information regarding the heterogeneity of glycosylation, including the modification sites and their linked glycan structures, which is useful for the functional study of target proteins. More importantly, this method does not require additional sample processing. However, as mentioned by Dang et al., the effectiveness of the method may be impacted by multiple parameters, such as glycopeptide structures (Dang et al., 2019). It also places greater requirements on the MS analyzer, and the profiling coverage of the glycosylation is limited because sufficient information regarding the peptides and the glycans in the MS2 spectra must be obtained for identification.

We summarize the advantages and disadvantages of each method mentioned above in Table 2 to help researchers to make appropriate choices according to the laboratory instrumentation and conditions.

**Table 2:** A comparison of different approaches for bisecting GlcNAc characterization based on MS detection.

**Detected target**	**Method**	**Criteria**	**Advantages**	**Disadvantages**
Glycan	β1,4-galactosyltransferase reaction	The relative abundance ratios of pairs of glycans varying in composition by a single GlcNAc unit were not significantly altered	1. Easy to process; 2. Easy to make a comparison.	1. Not easy to quantify; 2. Requires an extra enzymatic treatment.
	GC-MS	The presence of 3,4,6-linked mannose	1. Easy to quantify;	1. Need to perform PMAA derivatization; 2. Requires GC-MS instrumentation.
	MSn (n > 2)	The presence of the fragment ion m/z 444	1. Does not require extra sample processing; 2. Can select target glycans if required.	1. High requirements for the mass spectrometer; 2. High requirements for the operators
Glycopeptide	MS2	The presence of [Pep+HexNAc3Hex] or [Pep+FucHexNAc3Hex] or both	1. Does not require extra sample processing; 2. Can select target glycopeptides if required.	1. High requirements for the mass spectrometer; 2. The effectiveness may be affected by multiple parameters, such as glycopeptide structures.

## Synthesis of Bisecting Glycans

With more studies focusing on the special bisecting glycans, the importance of this type of glycan in cell biology has been discovered. Indeed, glycosylation modification plays an important role in protein functions due to participation in the functional domain of protein configuration (Luber et al., 2018). It has been reported that human IgG, an important immune system molecule, possesses glycans containing bisecting GlcNAc (Le et al., 2016; Lu and Holland, 2019; Shade et al., 2019). Thus, only synthesizing the sequence of proteins, but not the glycan chains, is insufficient for protein function. Syntheses of glycans or glycoproteins containing bisecting GlcNAc structures have been reported in many papers (Wang et al., 2009; Castilho et al., 2011; Luber et al., 2018; Manabe et al., 2018; Yang et al., 2018). Synthesis of glycans or glycoproteins containing bisecting GlcNAc structures is helpful for glycomic and glycoproteomic research and will improve the development of protein-based therapeutics and the generation of glycan-engineered therapeutic antibodies (Castilho et al., 2011, 2015).

In 2007, Unverzagt et al. reported the first chemical synthesis of highly branched pentaantennary N-glycans and derivatives with bisecting GlcNAc modifications (Eller et al., 2007). The chemical synthesis of a bisecting GlcNAc could also be achieved through [4+2] and [6+2] glycosylations. This synthetic method reduces the number of reaction steps but faces two difficulties, namely, low yields and poor synthesis selectivity for key glycosylations (Manabe et al., 2018). A modular synthesis of 16 cores of mammalian complex-type N-glycans with optional core fucose and bisecting GlcNAc has been established by Unverzagt et al., and core fucosylated and bisected N-glycans could be synthesized with unprecedented efficiency and purity by integrating a one-pot protocol (Luber et al., 2018).

Biosynthesis of glycoproteins with bisecting GlcNAc glycans has been performed in glycoengineered *Nicotiana benthamiana*, which lacks plant-specific N-glycosylation (Castilho et al., 2011) but expresses a modified version of human GlcNAc-T III. However, GlcNAc-T III is sometimes not very active when fused to the Golgi α-mannosidase II-cytoplasmic tail, transmembrane domain, and stem (GMII-CTS) region. Therefore, more studies are required to overcome these difficulties.

## Conclusions

Researchers are now beginning to realize the importance of bisecting GlcNAc glycans. We reviewed its importance in neurological systems, immune tolerance, IgG, and tumor metastasis and development and then introduced a series of MS approaches for bisecting GlcNAc detection. Compared to the traditional lectin recognition method, MS-based methods can be quantifiable, can target the glycan and glycopeptide of interest, and can provide details of the glycosylation sites and glycan components. In addition, MS approaches are more sensitive, and limits on sample amounts are overcome in glycosylation studies. However, there are bottlenecks in the use of current MS technology to detect the bisecting GlcNAc. The sensitivity of MS detection to glycosylation modification is still limited, and thus specific enrichment of the glycans or glycopeptides is needed. Especially for the MSn analysis, only glycans with higher abundance could be interpreted in detail and with accuracy. In addition, the construction of high-quality MSn spectral databases as well as an understanding of fragmentation mechanisms are also vital for developing the *in silico* fragmentation tools. Precise prediction of bisecting GlcNAc will be achieved via developing a probabilistic generative model for the CID/HCD fragmentation by machine learning techniques. This review will be valuable for those researchers who are interested in the importance of bisecting GlcNAc in cell biology and can conduct studies in this field and will be helpful for advancing our understanding of bisecting GlcNAc.

## Author Contributions

QC participated in the discussion and drafted the manuscript. YR participated in the discussion and made corrections to the manuscript. ZT and FG participated in the discussion and made corrections to the manuscript. All authors have checked and approved the final manuscript.

## Conflict of Interest

The authors declare that the research was conducted in the absence of any commercial or financial relationships that could be construed as a potential conflict of interest.
